# Which Dendrimer to Attain the Desired Properties? Focus on Phosphorhydrazone Dendrimers†

**DOI:** 10.3390/molecules23030622

**Published:** 2018-03-09

**Authors:** Anne-Marie Caminade, Jean-Pierre Majoral

**Affiliations:** 1 CNRS, LCC (Laboratoire de Chimie de Coordination), 205 route de Narbonne, BP 44099, F-31077 Toulouse CEDEX 4, France; jean-pierre.majoral@lcc-toulouse.fr; 2 LCC-CNRS, Université de Toulouse, CNRS, F-31077 Toulouse CEDEX 4, France

**Keywords:** dendrimers, chemical composition, biological properties, phosphorhydrazone

## Abstract

Among the six Critical Nanoscale Design Parameters (CNDPs) proposed by Prof. Donald A. Tomalia, this review illustrates the influence of the sixth one, which concerns the elemental composition, on the properties of dendrimers. After a large introduction that summarizes different types of dendrimers that have been compared with PolyAMidoAMine (PAMAM) dendrimers, this review will focus on the properties of positively and negatively charged phosphorhydrazone (PPH) dendrimers, especially in the field of biology, compared with other types of dendrimers, in particular PAMAM dendrimers, as well as polypropyleneimine (PPI), carbosilane, and p-Lysine dendrimers.

## 1. Introduction

Prof. Donald A. Tomalia created the word “dendrimer” from two Greek words δέντρο (dendro), which translates to “tree”, and μέρος (meros), which translates to “part,” and synthesized the famous PAMAM (PolyAMidoAMine) dendrimers [1,2,3]. In addition to this pioneering work, he has recently proposed a new concept for unifying and defining nanoscience, which he has called “CNDPs,” which stands for Critical Nanoscale Design Parameters [4,5]. This concept can be applied to both hard (metal-based) nanoparticles and to soft (organic) nanoparticles. It is particularly well adapted to the definition and properties of dendrimers, which are soft nano-objects, synthesized step by step to finely tune their properties [6,7]. Six parameters have been defined in the CNDP concept; they concern the (*i*) size; (*ii*) shape; (*iii*) surface chemistry; (*iv*) flexibility/rigidity; (*v*) architecture; and (*vi*) elemental composition of nano-objects. In this review, we will particularly emphasize the sixth parameter, concerning the elemental composition of dendrimers, with particular attention on the differences this criterion induces on properties, despite identical terminal functions.

**Figure 1:**
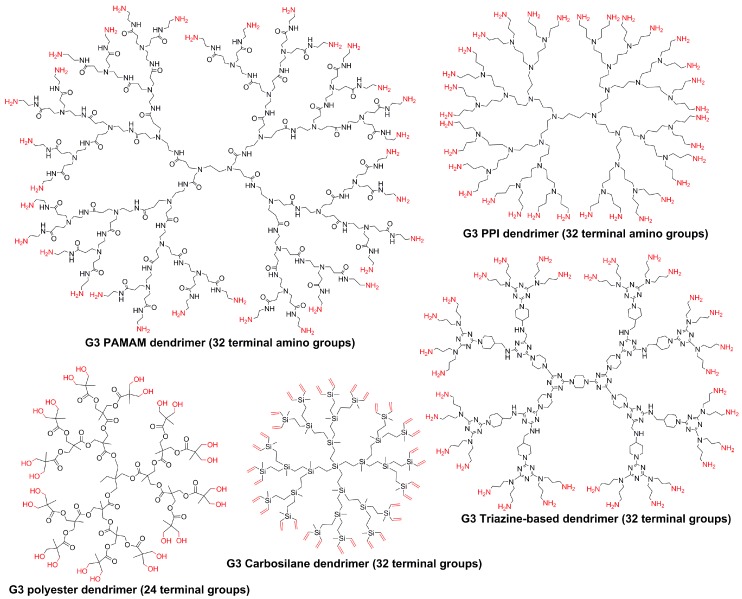
Chemical structure of different types of third-generation dendrimers.

A non-exhaustive search in the literature reveals that comparison experiments have been carried out in most cases with PAMAM dendrimers compared to other types of dendrimers, such as PPI (polypropyleneimine) [8,9,10], triazine [11,12], aliphatic ester [13,14], and carbosilane [15,16] dendrimers. The structure of the third generation of these dendrimers is shown in Figure 1. We will consider only publications in which comparative experiments have been done under conditions that are exactly the same and have been reported in an experimental publication, and not publications in which the comparison has been done with references to literature. Comparisons between PAMAM and PPI have been the most widely studied and in different areas. Differences in the fluorescence of the dye phenol blue encapsulated within the dendrimers demonstrated as expected that the interior of PPI dendrimers is slightly less polar than that of PAMAM dendrimers, both having amino terminal functions [17]. The comparison has also been carried out for catalysis. Different generations of both families of dendrimers have been used for the synthesis of gold nanoparticles (about 2 nm diameter in all cases) by a wet chemical NaBH_4_ method. The nanoparticles were then used for catalyzing the reduction of 4-nitrophenol. For Generations 2 and 3, it was shown that the rate constant with nanoparticles entrapped inside PAMAM dendrimers is higher than with PPI dendrimers, but no marked difference was observed for higher generations [18]. Generations 1–5 of PAMAM and PPI (called DAB in this study) dendrimers have been functionalized by promesogenic units derived from salicylaldimine. All these dendrimers exhibit liquid crystalline properties. The only differences between both series are a higher thermal stability and a wider mesophase temperature range in the PAMAM series, as a consequence of an increased rigidity, due to hydrogen bonds between the amido groups [19].

However, the largest number of comparisons between PAMAM and PPI dendrimers concerns their biological properties. Their toxicity has been tested toward the B16F10 cancerous cell line and in vivo in mice bearing this tumor. It has been shown that both families of dendrimers behaved essentially similarly, depending on the type of their terminal functions, and not on their internal structure [20]. Other toxicity assays have been carried out with Chinese hamster ovary and human ovarian carcinoma (SKOV3) cells. It has been shown that the two Generation 4 dendrimers with amino terminal functions are very harmful toward both types of cells [21]. MRI (magnetic resonance imaging) contrast agents based on gadolinium complexes have been grafted on the surface of Generation 4 PAMAM and PPI dendrimers, and these compounds were injected to mice. It was shown that the PPI dendrimer (DAB), compared with the PAMAM dendrimer, accumulated more significantly in the liver than in the blood [22]. Hyperpolarized xenon, generally protected in a cage of type cryptophane-A, is another MRI agent. These cages were entrapped most efficiently inside PAMAM dendrimers than inside PPI dendrimers (11 cages versus 4 for the fifth generations) [23]. Different types of molecules of biological interest have been entrapped also inside both families of dendrimers. This comprises the encapsulation of Vitamins C, B-3, and B-6 [24], phenylbutazone (an anti-inflammatory agent) for which PAMAM dendrimers have a higher loading ability than PPI dendrimers [25], and bortezomib (a proteasome inhibitor), which was by far more efficiently solubilized in water by PPI dendrimers than by PAMAM dendrimers [26].

A few other types of dendrimers have been compared to PAMAM dendrimers. For instance, the catalytic efficiency of carbosilane dendrimers bearing SCS-pincer palladium complexes as terminal functions has been compared to that of PAMAM dendrimers bearing the same type of terminal functions. The PAMAM dendrimers were found to be superior, by showing a higher reaction rate and a higher linear/branched ratio, in the cross coupling reaction between vinyl epoxide and styrylboronic acid. In the auto-tandem catalysis of cynnamyl chloride, hexamethylditin, and 4-nitrobenzaldehyde, only small differences were observed in the efficiency of both families of dendrimers [27]. The effect of PAMAM dendrimers and of triazine dendrimers of comparable size and number of terminal functions, both families being capped with primary amines, was tested toward platelet aggregation, in human platelet-rich plasma. It was shown that triazine dendrimers provoked platelet aggregation less aggressively than PAMAM dendrimers did [28]. The cytotoxicity of a series of aliphatic polyester dendrimers and PAMAM dendrimers, both having alcohol terminal functions, was evaluated toward human cervical cancer (HeLa), acute monocytic leukemia cells (THP.1), and primary human monocyte-derived macrophages. The aliphatic polyester dendrimers were found to be less toxic than the PAMAM dendrimers, and more easily cleavable [29].

To conclude this introductory overview, it seems that the internal structure is of relative importance for the properties of dendrimers. However, in this review, in which phosphorhydrazone dendrimers are compared with other types of dendrimers (including PAMAM and carbosilane dendrimers), we will show that the internal structure of dendrimers may be of crucial importance, in particular when considering the biological properties.

## 2. Phosphorhydrazone Dendrimers Compared to Other Types of Dendrimers

Two different families of phosphorus-containing dendrimers have been compared with other types of dendrimers: those having positive charges (ammoniums) as terminal functions, and those having negative charges (phosphonates) as terminal functions. They will be presented in this order. In all cases, the comparison is focused on the biological properties [30,31], as these dendrimers are soluble in water [32].

### 2.1. Positively Charged Phosphorus Dendrimers

**Figure 2:**
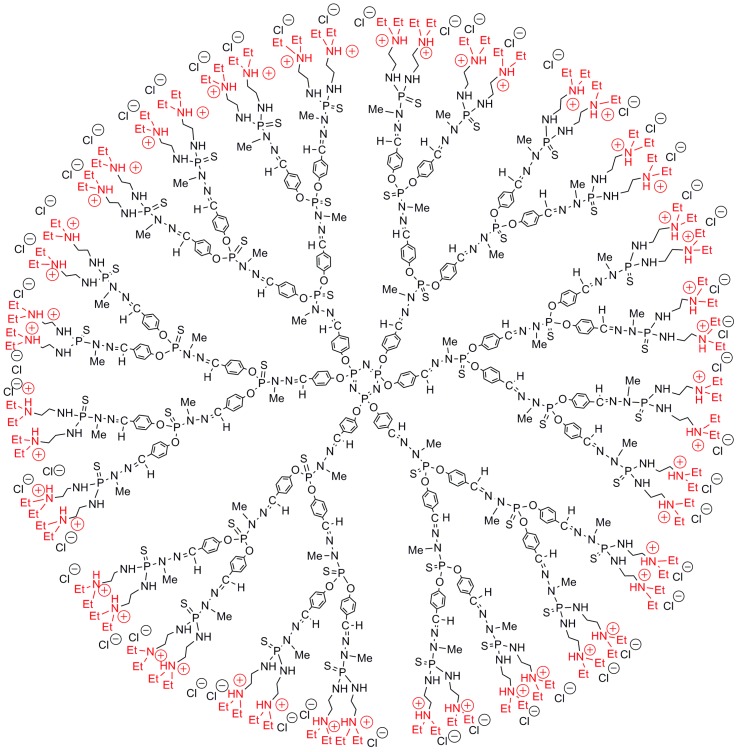
Water-soluble third-generation phosphorus dendrimer with 48 tertiary ammonium terminal functions.

Several generations of phosphorhydrazone (PPH) dendrimers having tertiary amines as terminal functions, subsequently protonated (the third generation is shown in Figure 2), have been compared essentially with PAMAM dendrimers, and occasionally with carbosilane dendrimers, having primary amines/ammoniums as terminal functions. These positively charged phosphorus dendrimers have been shown to be efficient transfection agents [33], and they display a high anti-prion activity in vivo, against the scrapie form of several strains of prions [34].

In the following sections, we will compare positively charged PPH dendrimers with other types of cationic dendrimers, concerning their interference with clinical chemistry tests, their efficiency as carriers, and their efficiency against neurodegenerative diseases.

#### 2.1.1. Comparative Interference with Clinical Chemistry Tests

Classical clinical chemistry tests (analysis of blood biochemical parameters) are widely used for assessing the toxicity of compounds. However, it is important to determine if the presence of nanoparticles in general and of dendrimers in particular can interfere or not with these tests. The tests were carried out with positively charged dendrimers of type phosphorhydrazone (Generation 4, 96 tertiary ammonium groups), PAMAM (Generation 4, 64 primary ammonium groups), and carbosilane (Generation 3, 24 quaternary ammonium groups) in standardized human serum, in the absence of cells. It was shown that these dendrimers interfere with the clinical chemistry tests, inducing changes in enzymes activity, and interactions with the test reagents (but not with a protein). These changes can be wrongly interpreted as the appearance of dysfunctions of the liver or buds, so this type of preliminary evaluation is necessary before any animal tests [35].

#### 2.1.2. Comparative Efficiency as Carriers

As already indicated, the transport and delivery properties of positively charged phosphorhydrazone dendrimers have been discovered very early, with the transport of the luciferase plasmid through the membrane of mammalian cells and its delivery inside the nucleus [33]. Changing the nature of the ammonium terminal functions did not improve the transfection efficiency [36]. Positively charged PAMAM and PPH dendrimers, both of Generation 4, were tentatively used to carry the plasmid, inducing an increased GDNF expression (the Glial cell line-Derived Neurotrophic Factor) into Schwann cells, isolated from sciatic nerves. The phosphorhydrazone dendrimers were found to be less effective than the PAMAM dendrimers for the transfection of these Schwann cells, but both were by far less effective than HIV-based lentiviruses. The transgenic Schwann cells were then used to regenerate transected peripheral nerves in rats [37]. PAMAM, PPH, and carbosilane dendrimers were used to complex different anticancer siRNA (small interfering RNA). Then, heparin was added to determine if the siRNA could be released from the dendrimer and if its structure was maintained. These dendrimers are effective for protecting siRNAs from RNase A activity, but treatment with heparin induced the release of siRNA only from the complexes obtained with PAMAM or carbosilane dendrimers, whereas the complexes formed with the phosphorhydrazone dendrimers were not destroyed by heparin [38]. These experiments were carried out in the perspective of the gene therapy of cancers, so these complexes were transfected in HeLa and HL-60 cancerous cell lines. The most effective carriers of siRNA among the three types of dendrimers tested were the PPH dendrimers [39].

#### 2.1.3. Comparative Efficiency against Neurodegenerative Diseases

The very first example in this field, using phosphorhydrazone dendrimers, concerned their interaction with the scrapie form of prions (PrP^Sc^), which is responsible for several types of spongiform encephalopathies, such as Creutzfeldt–Jakob disease and mad cow disease. The Generation 4 of positively charged phosphorhydrazone dendrimers was able to eliminate the PrP^Sc^ from infected cells, and was even found efficient in vivo, for mice infected with brain cells from terminally ill mice [34]. A sequel of this work concerned the interaction of dendrimers with the PrP 106–126 peptide, which is suspected to be involved in the formation of amyloid fibrils in these encephalopathies, as well as the Aβ 1–28 peptide for Alzheimer’s disease. The interaction of three types of positively charged dendrimers (phosphorhydrazone Generation 4, PAMAM Generations 5 and 6, and PPI Generation 3) with both types of peptides was assessed, using EPR analyzes with a spin probe. It was shown that the interactions of the dendrimers with PrP 106–126 are weaker than with Aβ 1–28. The PAMAM dendrimers seem to be better peptide-aggregation scavengers than the other dendrimers [40]. 

The interaction of the same three families of dendrimers with heparin, which is involved in the process of fibril formation in the prion diseases, was also measured. All these dendrimers interact with heparin, mainly by electrostatic interactions. These interactions are indirectly responsible for the inhibition or enhancement of fibril formation, depending on the concentration. At high concentrations, the dendrimers directly impede fibril formation, whereas at low concentrations, they sequester the heparin, preventing it from inducing fibril formation. The dye Thioflavin T-3516 (ThT), which is generally used for detecting amyloid structures, as it fluoresces only in their presence, was used for detecting the interaction of the phosphorhydrazone dendrimers with heparin. Although ThT did not fluoresce in the presence of the dendrimers alone, or heparin alone, a fluorescence was detected for the complex between heparin and the phosphorhydrazone dendrimers. Only these phosphorus dendrimers behaved this way, as no fluorescence was detected for the complexes formed with PAMAM or PPI dendrimers [41].

Rotenone is a pesticide, which is also a damaging agent, increasing the amount of reactive oxygen species (ROS) in neurons, α-synuclein aggregation, and the activation of microglia, and which is associated with an increased risk of Parkinson’s disease. In view of the above-mentioned properties of dendrimers on brain diseases, in particular for preventing aggregation and the formation of fibrils, it seemed important to investigate if positively charged dendrimers can prevent the damages caused by rotenone on mouse mHippoE-18 cells in vitro. The dendrimers tested here were PAMAM dendrimers, PPH dendrimers, and small viologen-phosphorus dendrimers [42,43]. These dendrimers increased cell viability, decreased ROS production, and preserved the mitochondrial function [44].

### 2.2. Negatively Charged Phosphorus Dendrimers

**Figure 3:**
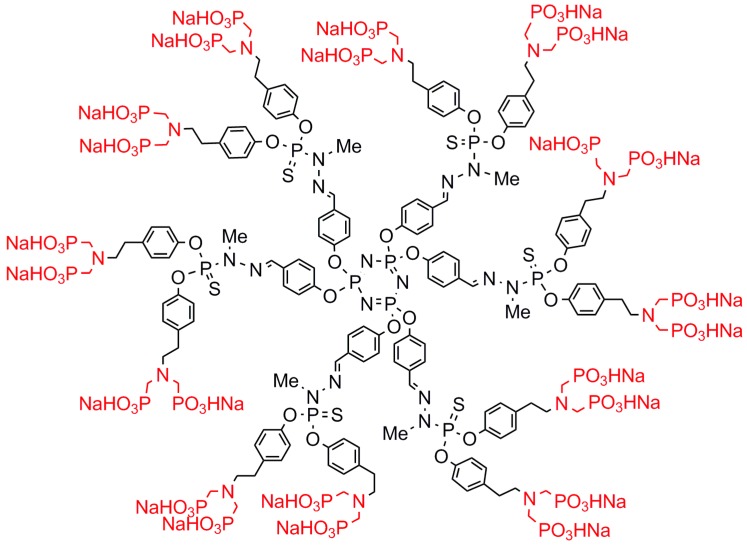
First-generation phosphorhydrazone dendrimer with azabisphosphonate terminal functions (ABP).

Negatively charged dendrimers are classically obtained by grafting carboxylic acids as terminal functions, from which sodium salts are easily obtained. This was done in particular with phosphorhydrazone dendrimers [45,46]. However, the negatively charged phosphorus dendrimer possessing the most important biological properties up to now has not carboxylates but azabisphosphonate salts as terminal functions. The structure of the first generation is shown in Figure 3, called “ABP,” which stands for AzaBisPhosphonate. In a first experiment, it was shown that this dendrimer is able to induce in vitro the activation of human monocytes, which are a pivotal cell population of innate immunity in the blood [47]. It was shown later that this activation of monocytes occurs through an anti-inflammatory pathway [48]. Among a series of PPH dendrimers having different types of negatively charged terminal functions and of different generations (0 to 2), it was shown that the first generation shown in Figure 3 was the most active [49]. Tailoring the number of terminal functions from 2 to 30 for first-generation PPH dendrimers, by playing with the reactivity of the cyclotriphosphazene, demonstrated that compounds decorated with 8–12 azabisphosphonate terminal functions are the most efficient [50].

In a second experiment, it was shown that the same dendrimer ABP is able to multiply by several hundreds the number of natural killer (NK) cells, which are pivotal for innate immunity, implicated in the early immune response against infections and playing a crucial role in anticancer immunity. As the proliferation of NK cells was extremely tedious to achieve before our work, it was important to verify if the NK cells obtained thanks to this dendrimer were fully functional. Their ability to kill the same cancer cell lines with the same efficiency as uncultured NK cells was succesfully assessed with respect to 15 cell lines (leukemia and carcinoma). Importantly, no agressiveness of the NK cells generated with this dendrimer toward lymphocytes coming from the same blood donor was observed, demonstrating the safety of this compound [49]. It was shown later on that a multistep cross-talk between monocytes and NK cells is necessary before the proliferation of NK cells [51].

In a third experiment, the anti-inflammatory properties of this dendrimer ABP were tested in vivo against chronic inflammatory diseases such as multiple sclerosis (MS) in mice. MS is a chronic inflammatory disease of the central nervous system, thought to be due to an inflammatory attack by autoreactive T cells, which amplify an inflammatory cascade, inducing myelin sheath, resulting in impaired nerve conduction. In a mouse model of MS, in which an experimental autoimmune encephalomyelitis (EAE) has been induced, the dendrimer ABP prevents the development of EAE, and inhibits the progression of established disease. One important mechanism of action of the dendrimer ABP in this case is that it skews the cytokine production by splenocytes from an inflammatory pattern to an anti-inflammatory one [52]. 

In continuing the study of the structure/activity relationship, the same terminal functions were grafted to the surface of a series of dendrimers. These functions were first grafted to the surface of a first-generation PPI dendrimer, and both dendrimers were tested against another chronic inflammatory disease, rheumatoid arthritis (RA). RA is an autoimmune inflammatory disease, which is characterized by inflammation of the synovial membrane, cartilage degradation, and bone erosion, leading to major handicaps. The ABP dendrimer was found to be very efficient in mice suffering from an RA-like inflammatory disease, whereas the PPI dendrimer had no activity. The dendrimers were given weekly, either intravenously or orally. For mice treated with the dendrimer ABP, normal synovial membranes, reduced levels of inflammatory cytokines, and the absence of both cartilage destruction and bone erosion were observed. Dendrimer ABP increases the level of anti-inflammatory cytokines and has anti-osteoclastic properties. On the contrary, for mice that received the PPI dendrimer decorated with the same azabisphosphonate terminal functions, no difference was observed compared to untreated mice [53]. 

**Figure 4:**
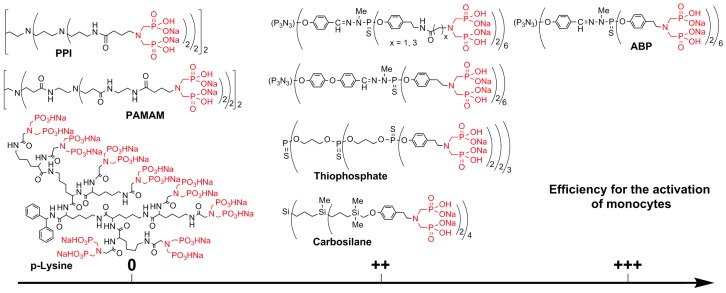
Efficiency of the activation of monocytes, depending on the internal structure of the dendrimers (0: no activation; ++: good activation; +++: the highest activation).

This work displayed for the first time a drastic difference between the biological activity of two dendrimers having the same terminal functions, but a different internal structure. This idea was then developed to test a larger number of dendrimer families. As the activation of monocytes is the first step for all biological properties of the dendrimer ABP, this was considered as the suitable test to determine the properties of these dendrimers (Figure 4). Dendrimers of type thiophosphate and carbosilane were functionalized with exactly the same function as with ABP. Dendrimers with amine terminal functions (PPI, PAMAM, and p-Lysine) were functionalized by peptide couplings, affording a linker different from the one used for ABP. Thus, the same linker was used also on the surface of a first-generation phosphorhydrazone dendrimer. The dendrimers containing heteroatoms (P or Si) in their structure, even those having a structure very different from that of ABP (thiophosphate and carbosilane), are efficient for the activation of monocytes, even if ABP is still the most efficient. On the contrary, all the “organic” dendrimers (PPI, PAMAM, and p-Lysine) have absolutely no efficiency for the activation of monocytes. In order to try to understand this surprising result, all-atom molecular dynamics simulations were carried out for all of these families of dendrimers. It was shown that all of the compounds that are active have all of their terminal functions gathered in a single side of the dendrimers, which look like cauliflowers and afford a localized high density of functions. On the contrary, the dendrimers that are non-active have a rather spherical structure, and the terminal functions are distributed all over the surface, affording a low local density of functions. This study was the largest given the number of different families that were assayed in identical conditions [54].

## 3. Conclusions

In view of all these results, how can our initial question of which dendrimer attains the most desirable properties be answered? Concerning positively charged dendrimers, in particular their transfection efficiency when using plasmids, the PAMAM dendrimers are generally more efficient than the phosphorhydrazone dendrimers. However, when considering the delivery of siRNA, the phosphorhydrazone dendrimers seem more efficient than the PAMAM dendrimers. For other properties, in particular concerning brain diseases, PAMAM, PPI, and PPH dendrimers have almost the same properties, with either PAMAM or PPH being slightly better depending on the precise type of experiment. The situation is very different concerning negatively charged dendrimers. Indeed, with strictly identical terminal functions, the dendrimers containing heteroatoms (P or Si) in their structure have anti-inflammatory properties, whereas the “organic” dendrimers do not. Table 1 summarizes the types of dendrimers and their types and numbers of terminal functions, which have been compared to PPH dendrimers.

Thus, the real conclusion of this review is that the sixth parameter of the CNDPs, concerning the elemental composition of nano-compounds, especially dendrimers, has to be taken into account when dealing with properties, especially biological properties. Definitively, the internal structure of dendrimers is not an “innocent” scaffold.

## Figures and Tables

**Table 1:** Types of dendrimers, with the nature and number of their terminal functions, used for comparison in different biological experiments. The most efficient compound for each experiment is highlighted in red.

PPH	PAMAM	PPI	PCSi	P-Lys	Experiment	Ref.
-NEt_2_H)_96_	-NH_3_)_64_		-NMe_3_)_24_		Clinical tests	[35]
-NEt_2_H)_96_	-NH_3_)_64_				Transfection	[37]
-NEt_2_H)_48_/ -NEt_2_H)_96_	-NH_3_)_32_/ -NH_3_)_64_		-NMe_3_)_8_		Protection SiRNA ^1^	[38]
-NEt_2_H)_48_/ -NEt_2_H)_96_	-NH_3_)_32_/ -NH_3_)_64_		-NMe_3_)_8_		Carrier of Si RNA	[39]
-NEt_2_H)_96_	-NH_3_)_64_/ -NH_3_)_128_	-NH_3_)_16_			Peptide aggregation scavenger	[40]
-NEt_2_H)_96_	-NH_3_)_64_/ -NH_3_)_128_	-NH_3_)_16_			Interaction with heparin	[41]
-NEt_2_H)_48_/ -NEt_2_H)_96_	-NH_3_)_32_/ -NH_3_)_64_				Decrease ROS ^2^ levels	[44]
(PO_3_HNa)_2_]_12_		(PO_3_HNa)_2_]_8_			Against RA ^3^	[53]
(PO_3_HNa)_2_]_12_^4^	(PO_3_HNa)_2_]_8_	(PO_3_HNa)_2_]_8_	(PO_3_HNa)_2_]_8_	(PO_3_HNa)_2_]_8_	Activation of monocytes	[54]

^1^ Small interfering RNA. ^2^ Reactive Oxygen Species. ^3^ Rheumatoid Arthritis. ^4^ Same efficiency with PPH (PO_3_HNa)_2_]_8_ [50].
